# A Two-Hop mmWave MIMO NR-Relay Nodes to Enhance the Average System Throughput and BER in Outdoor-to-Indoor Environments

**DOI:** 10.3390/s21041372

**Published:** 2021-02-16

**Authors:** Randy Verdecia-Peña, José I. Alonso

**Affiliations:** 1 Information Processing and Telecommunications Center, Universidad Politécnica de Madrid, 28040 Madrid, Spain; ignacio@gmr.ssr.upm.es; 2 ETSI de Telecomunicación, Universidad Politécnica de Madrid, 28040 Madrid, Spain

**Keywords:** millimeter-wave, new radio amplify-and-forward, new radio decode-and-forward

## Abstract

Millimeter-Wave (mmWave) bands are receiving enormous attention in 5G mobile communications, due to the capability to provide a multi-gigabit transmission rate. In this paper, a two-hop architecture for 5G communications with the capacity to support high end-to-end performance due to the use of Relay Nodes (RNs) in mmWave-bands is presented. One of the novelties of the paper is the implementation of Amplify-and-Forward (A&F) and Decode-and-Forward (D&F) RNs along with a mmWave-band transceiver chain (Tx/Rx). In addition, two approaches for channel estimation were implemented at the D&F RN for decoding the backhaul link. One of them assumes complete knowledge of the channel (PCE), and the other one performs the channel estimation through Least Square (LS) estimator. A large number of simulations, using MATLAB${}^{\mathrm{TM}}$ and Simulink${}^{\mathrm{TM}}$ software, were performed to verify the potential benefits of the proposal two-hop 5G architecture in an outdoor-to-indoor scenario. The main novelty in performing these simulations is the use of signals with 5G features, as DL-SCH transport channel coding, PDSCH generation, and SS Burst generation, which is another of the main contributions of the paper. On the other hand, mmWave transmitter and receiver chains were designed and implemented with off-the shelf components. The simulations show that the two-hop network substantially improves the Key Performance Indicators (KPIs), Bit Error Rate (BER), and Throughput, in the communications between the logical 5G Radio Node (gNodeB), and the New Radio User Equipment (NR-UE). For example, a throughput improvement of 22 Mbps is obtained when a 4 × 4 × 2 MIMO D&F with LS architecture is used versus a SISO D&F with PCE architecture for Signal-to-Noise Ratio (SNR) = 20 dB and 64-QAM signal. This improvement reaches 96 Mbps if a 256-QAM signal is considered. The improvement in BER is 11 dB and 10.5 dB, respectively, for both cases. This work also shows that the obtained results with D&F RNs are better than with A&F RNs. For example, an improvement of 17 Mbps in the use of SISO D&F with LS vs. SISO A&F, for the 64-QAM signal is obtained. Besides, this paper constitutes a first step to the implementation of a mmWave MIMO 5G cooperative network platform.

## 1. Introduction

Today, the number of people demanding fast and efficient data speeds has increased exponentially. In this sense, there is a constant evolution towards networks with data speeds of 10 Gbps [1,2], which will allow the development of new applications, such as HD video and online games [3]. The New Generation Mobile (5G) wireless communication systems are called to provide 1000-times more capacity and support 100-times more smart devices than the current Fourth Generation (4G) [4]. In this context, Massive MIMO is one of the key enabling technology for new 5G networks [5]. Massive MIMO systems enhance cellular spectral efficiency and provide sufficient spatial degrees of liberty for multiplexing several data streams rendering to serve multiple users simultaneously.

Millimeter-Wave (mmWave) spectrum [6] is emerging as another enabling technology in 5G networks. It has been a topic of interest in recent years by academia, standards organizations, regulatory commissions, and industry, and it is a key candidate for addressing the challenges of bandwidth shortage for 5G systems. However, signals at mmWave bands undergo severe path loss and are highly sensitive to blockage as compared to FR1 frequency bands of 5G. On the other hand, thanks to smaller wavelengths of mmWave allowing more antennas to pack within the same physical area, this drives to use of a large number of antennas at the transceivers, thereby forming massive MIMO systems. In conclusion, the combination of the massive MIMO and mmWaves emerge as a concrete tool to address the requirements of 5G network deployments [7]. However, the hardware implementation of mmWave Massive MIMO systems present significant technological challenges [8]. The common solution for their implementation is the use of Planar Phased Array antennas. Nevertheless, these systems present the problem of mutual coupling between antennas. Some solutions and various isolation improvement mechanisms have been proposed [9,10]. In our case, horn antennas were used in the mmWave transceiver chain, which, at the cost of reducing the couplings between antennas, limit the massive MIMO capabilities of the system.

In the last few years, Relay Node (RN) protocols have been a research topic that has attracted much interest from researchers. In fact, relaying technology has proven to be an optimal solution to guarantee the quality of service in certain scenarios. In this context, the use of RNs presents several advantages, such as a reduction in total transmission power, a decrease in network capacity dispersion, the elimination of penetration losses in outdoor-to-indoor scenarios, and an increase in network coverage that combined with the access link in millimeter bands can lead to a notable improvement in network operation [11,12]. Furthermore, RN techniques have been studied and considered for standardization by the 3GPP [13]. The most common strategies are Amplify-and-Forward (A&F) and Decode-and-Forward (D&F) [14], which have been considered in the LTE technology together mmWave MIMO techniques. However, the mmWave MIMO RNs have not been deeply studied in the new scenarios proposed by 3GPP [15]. In these scenarios, 5G New Radio (NR) Networks using RNs [16] are expected to provide higher performance and solve some of the issues for deployment in these scenarios.

Motivated by the above issues, in this paper, we implemented a mmWave MIMO Relay cooperative network, where signals with 5G features were considered. The major contributions of this paper are summarized as follows:

- A two-hop architecture using out-of-band RNs in mmWave frequencies and with MIMO capabilities is presented. In the simulated architecture, the NR 5G signal features: DL-SCH transport channel coding, PDSCH generation, and SS Burst generation, were used. The relay assists the communication in the outdoor-to-indoor environment between the gNodeB and User Equipment (UE).
- Two relaying strategies, Amplify-and-Forward (A&F) and Decode-and-Forward (D&F) were implemented. Besides, two alternatives of D&F protocol were considered. The first takes into account the channel knowledge and the second performs the channel estimation through the Least Square (LS) estimator and Bi-Cubic Interpolate (BCI), both in the Backhaul Link of the architecture proposal.
- Analytical expressions for both A&F and D&F relaying strategies of the proposed two-hop mmWave MIMO Relay cooperative system were derived.
- A mmWave transmitter and receiver chains were designed using off-the shelf components. Furthermore, extensive simulations were taken into account and carried out with the Mathlab${}^{\mathrm{TM}}$ and Simulink${}^{\mathrm{TM}}$ tools, considering the non- linearity of mmWave subsystems.

The remainder of this paper is organized as follows. In Section 2, the system model of the two-hop MIMO mmWave NR-RN is presented. Then, the network model of the mmWave NR-RN cooperative system in an outdoor-to-indoor scenario is detailed in Section 3, as well as the implementation of the Decode-and-Forward NR-RN and the mmWave transmitter and receiver chains. In Section 4, the numerical results of the designed architecture are presented and discussed. Conclusions are drawn in Section 5.

## 2. System Model and Basic Assumptions

### 2.1. Model Description

**Figure 1:**
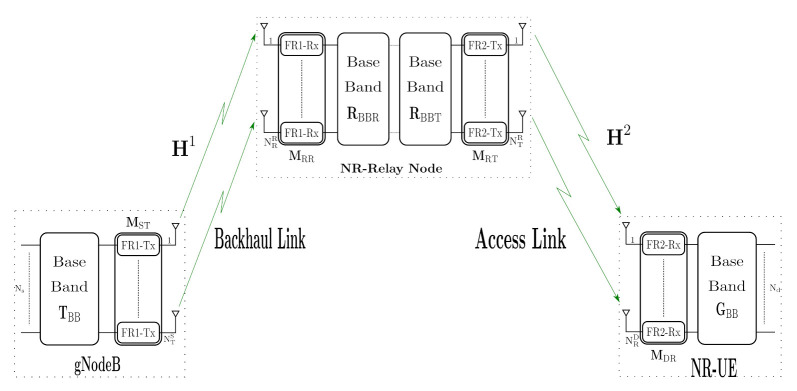
MIMO Millimeter-Wave (mmWave) downlink New Radio (NR)-Relay Node (RN) structure.

In this section, the two-hop mmWave MIMO NR RN cooperative architecture, shown in Figure 1, will be presented and described. It comprises a gNodeB, a NR-RN and a NR-User Equipment (NR-UE) working in a Half-Duplex (HD). a) The gNodeB includes two blocks: the baseband signal processing block and array of RF amplifiers chains to up-convert the signal to 5G FR1 (<6 GHz) frequency band (FR1-Tx). In our architecture, these signal processing and up-conversion stages were implemented using Universal Software Radio Peripheral (USRP). b) The other important block of the system is the NR-RN. Its function is to convert the 5G FR1 (<6 GHz) frequency band (Backhaul Link -FR1-) to mmWave bands (Access Link -FR2-). It comprises an array of receiver chains in 5G FR1 band (FR1-Rx), emulated by USRPs, a baseband signal processing block for the implementation of the signal processing algorithms associated with the implemented relaying strategies ( $R_{\mathrm{BBT}}$ and $R_{\mathrm{BBR}}$ matrices, in Section 2.2 and Section 2.3), and a mmWave conversion stage (FR2-Tx) and c) finally, the third block, NR-UE, which consists of an array of mmWave receiver chains (FR2-Rx), which receives the signal from the FR2-Tx chains through the access link, and down-convert it for further processing in the baseband block ($G_{\mathrm{BB}}$). The direct link between the gNodeB and the UE is not studied because it is considered to operate in different band frequencies.

We assume that at the gNodeB, the number of transmit antennas is $N_{T}^{S}$, the number of data symbols is $N_{s}$, and the number of FR1 transmitters (FR1-Tx) is equal to $N_{T}^{S}$. From Figure 1, the RN is equipped $N_{R}^{R}$ receive antenna in which corresponding with the number of FR1 receiver (FR1-Rx), and RF mm-Wave chains (FR2-Tx) and number of the transmitter antennas, both equal to $N_{T}^{R}$. In addition, in the destination, the number of FR2 receiver (FR2-Rx) depends to the number of receiver antennas $N_{R}^{D}$.

**Figure 2:**
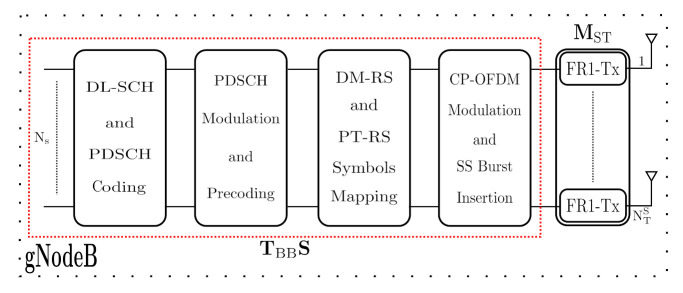
Baseband gNodeB structure.

We consider downlink cooperative SU-MIMO system using precoding in the gNodeB, RN, and UE. To enable precoding, we assume that the gNodeB and RN has full channel side information knowledge. In fact, the precoding process in the source can be represented by the product between the RF transmitter, $M_{ST}$∈$C^{N_{T}^{S}\times N_{T}^{S}}$, and the baseband precoding matrix, $T_{\mathrm{BB}}$∈$C^{N_{T}^{S}\times N_{s}}$. The source transmits S∈$C^{N_{s}\times T}$ data symbols, where T is the number of used subcarrier for the transmission. Therefore, the transmitted signal from the source can be expressed by

(1) $$X=M_{ST}T_{\mathrm{BB}}S,$$

where power normalization is satisfied such that $\left|\right|M_{T}^{S}T_{\mathrm{BB}}{\left|\right|}_{F}^{2}=TN_{T}^{S}$. The transmitted signal (1) from gNodeB is implemented through the gNodeB structure explained in Figure 2, where the data symbols ($N_{s}$) are coded through the DL-SCH and PDSCH Coding block. After that, over PDSCH is performed the modulation (depend on the implemented M-QAM constellation) and the precoding stage. Then, reference signals are mapped and CP-OFDM modulation is performed. Finally, it is inserted a portion of SS Burst and the baseband signal is upconverted to the FR1 band by $M_{\mathrm{ST}}$. It should be noticed that the generated and configuration of the signal and channels in the gNodeB fulfill the 5G NR standardization of the 3GPP, which is one of the main novelty of our proposal.

### 2.2. A&F NR-RN Signal Model

In this subsection, we consider the two-hop MIMO A&F NR-RN configuration from Figure 1. Then, let X denotes the symbols matrix to be transmitted by gNodeB. In the first-phase, the received signal at the relay is represented as

(2) $$Y_{\mathrm{AF}}^{1}=H^{1}X+N_{\mathrm{AF}}^{1},$$

where $H^{1}$∈$C^{N_{R}^{R}\times N_{T}^{S}}$ denotes a source-to-relay node FR1 (<6 GHz) channel matrix. $N_{\mathrm{AF}}^{1}$∈$C^{N_{R}^{R}\times T}$ indicates noise matrix with independent and identical distributed random variables $N∼(0,\sigma_{N_{\mathrm{AF}}^{1}}^{2})$, where $\sigma_{N_{\mathrm{AF}}^{1}}^{2}$ denotes the noise variance. The signal $Y_{\mathrm{AF}}^{1}$ is received through FR1-Rx chains $M_{RR}$∈$C^{N_{R}^{R}\times N_{R}^{R}}$ at the relay. Then, it is precoded by precoder matrices $R_{\mathrm{BBR}}$∈$C^{N_{R}^{R}\times N_{R}^{R}}$ and $R_{\mathrm{BBT}}$∈$C^{N_{R}^{R}\times N_{R}^{R}}$, and RF chains $M_{\mathrm{RT}}$∈$C^{N_{T}^{R}\times N_{R}^{R}}$. In fact, the product $R_{\mathrm{BBT}}R_{\mathrm{BBR}}=G_{\mathrm{AF}}$, which depicts a diagonal matrix that on your principal diagonal contains a gain vector ($g_{\mathrm{AF}}$). Considering the processing above described, the transmit signal at the A&F RN is expressed as

(3) $$X_{\mathrm{AF}}=M_{\mathrm{RT}}G_{\mathrm{AF}}M_{\mathrm{RR}}^{H}Y_{\mathrm{AF}}^{1},$$

where $M_{\mathrm{RR}}^{H}$ denotes the hermitian matrix of $M_{\mathrm{RR}}$. In the second-phase, the received signal at the UE can be written as

(4) $$Y_{\mathrm{AF}}^{2}=H^{2}X_{\mathrm{AF}}+N_{\mathrm{AF}}^{2},$$

where $H^{2}$∈$C^{N_{R}^{D}\times N_{T}^{R}}$ describes relay-to-destination mm-Wave channel matrix, and the matrix $N_{\mathrm{AF}}^{2}$ is the components respective of Additive White Gaussian Noise (AWGN), which ∈$C^{N_{R}^{D}\times T}$ with $N∼(0,\sigma_{N_{\mathrm{AF}}^{2}}^{2})$. At the destination node, the received signal (4) is processed by the RF matrix $M_{\mathrm{DR}}$∈$C^{N_{R}^{D}\times N_{R}^{D}}$ and baseband matrix $G_{\mathrm{BB}}$∈$C^{N_{d}\times N_{R}^{D}}$, which are equivalent to inverse processing performed by the gNodeB. The received signal is converted to baseband frequency, and the baseband signal and physical channels are demodulated and decoded, respectively. Finally, the estimated signal of the destination can be expressed as

(5) $$\begin{array}{cc} \hat{S} & =G_{\mathrm{BB}}^{H}M_{\mathrm{DR}}^{H}\left(H^{2}X_{\mathrm{AF}}+N_{\mathrm{AF}}^{2}\right)\\ \; & =G^{H}H^{2}M_{\mathrm{RT}}G_{\mathrm{AF}}M_{\mathrm{RR}}^{H}H^{1}TS+N_{D}\\ \; & =S+N_{D} \end{array},$$

where $G=G_{\mathrm{BB}}M_{\mathrm{DR}}$, $T=M_{\mathrm{ST}}T_{\mathrm{BB}}$, and $N_{D}=G^{H}\left(H^{2}M_{\mathrm{RT}}G_{\mathrm{AF}}M_{\mathrm{RR}}^{H}N_{\mathrm{AF}}^{1}+N_{\mathrm{AF}}^{2}\right)$. In (5), the first term is the desired signal and the second term is the noise, which denotes the noise in the backhaul and access links at the cooperative system, respectively.

### 2.3. D&F NR-RN Signal Model

**Figure 3:**
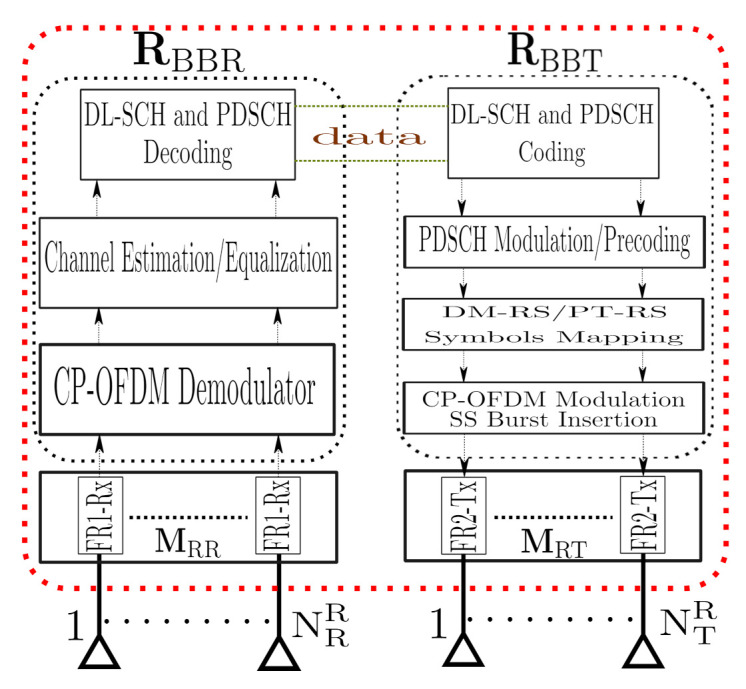
mmWave MIMO NR Decode-and-Forward (D&F) Relay Node (RN) structure.

In this subsection, the system model of the two-hop MIMO mm-Wave D&F NR-RN will be addressed. Taking into account Figure 1 and transmitted signal (1) from the source, in the backhaul link, the received data at the D&F protocol in a block can be expressed as

(6) $$Y_{\mathrm{DF}}^{1}=H^{1}X+N_{\mathrm{DF}}^{1},$$

where matrix $N_{\mathrm{DF}}^{1}$∈$C^{N_{R}^{R}\times T}$ with $N∼(0,\sigma_{N_{\mathrm{DF}}^{1}}^{2})$ represents the AWGN in the first-hop, where $\sigma_{N_{\mathrm{DF}}^{1}}^{2}$ is the noise variance. $H^{1}$∈$C^{N_{R}^{R}\times N_{T}^{S}}$ denotes the sub-6 GHz MIMO channel matrix between the source and relay. In the D&F RN, the signal (6) is captured through the matrix $M_{\mathrm{RR}}$∈$C^{N_{R}^{R}\times N_{R}^{R}}$, which in the real environment can be emulated by an SDR as has been proposed in Reference [17]. Worthy of highlighting is that D&F strategy performs two stages: firstly, decode the received signal, and, before forward the signal to the destination, it encodes the estimated symbols, as shown in Figure 3, which supposes the major difference in comparison with A&F protocol.

In fact, the decoding and encoding stages are performed by the matrices $R_{\mathrm{BBR}}$∈$C^{N_{s}\times N_{R}^{R}}$ and $R_{\mathrm{BBT}}$∈$C^{N_{T}^{R}\times N_{s}}$, respectively. Consequently, the transmitted signal by the RN can be given as

(7) $$\hat{X}=M_{\mathrm{RT}}R_{\mathrm{BBT}}\tilde{S},$$

where matrix $M_{\mathrm{RT}}$∈$C^{N_{T}^{R}\times N_{T}^{R}}$ denotes the transmitter RF mmWave chains of the RN. On the other hand, $R_{\mathrm{BBT}}$∈$C^{N_{T}^{R}\times N_{s}}$ is the baseband matrix and $\tilde{S}$ represents the estimated data symbols matrix at the D&F protocol. Taking into account (7), the received signal at the UE can be expressed by

(8) $$Y_{\mathrm{DF}}^{2}=H^{2}\hat{X}+N_{\mathrm{DF}}^{2},$$

where $H^{2}$∈$C^{N_{R}^{D}\times N_{T}^{R}}$ is the channel matrix of the access link (relay-to-destination link) and $N_{\mathrm{DF}}^{2}$∈$C^{N_{R}^{D}\times N_{T}^{R}}$ denotes the AWGN with $N∼(0,\sigma_{N_{\mathrm{DF}}^{2}}^{2})$, where $\sigma_{N_{\mathrm{DF}}^{2}}^{2}$ is the noise variance in the access link. Considering the data processing at the UE through $M_{\mathrm{DR}}$ and $G_{\mathrm{BB}}$, which the estimated received signal at the destination can be written as

(9) $$\begin{array}{cc} \hat{S} & =G_{\mathrm{BB}}^{H}M_{\mathrm{DR}}^{H}\left(H^{2}\hat{X}+N_{\mathrm{DF}}^{2}\right)\\ \; & =G^{H}H^{2}R_{\mathrm{RT}}\tilde{S}+N_{D}\\ \; & =\tilde{S}+N_{D} \end{array},$$

where $N_{D}=G^{H}N_{\mathrm{DF}}^{2}$, $G=G_{\mathrm{BB}}M_{\mathrm{DR}}$, and $R_{\mathrm{RT}}=M_{\mathrm{RT}}R_{\mathrm{BBT}}$. In (9), the first term is the desired signal and the second term is the noise, which represents the noise in access link at the Decode-and-Forward (D&F) cooperative system, respectively.

## 3. Network Model of the mmWave MIMO NR-Relay Node (RN) Cooperative System

**Figure 4:**
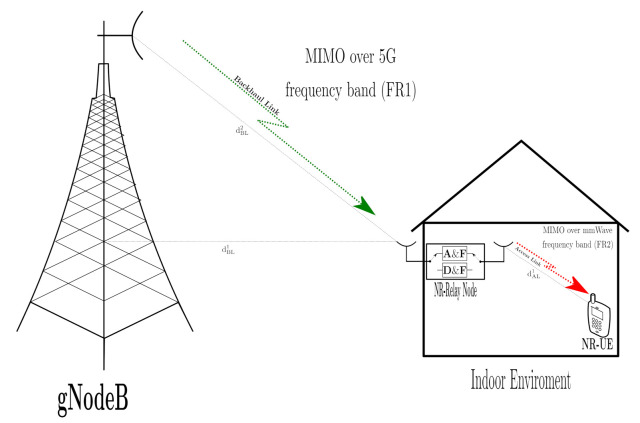
Outdoor-to-Indoor environment scenario with mmWave MIMO NR-RN.

In this section, the mmWave MIMO New Radio RN cooperative network is presented. The scenario is situated in an urban environment, specifically, outdoor-to-indoor, as is shown in Figure 4. A&F and D&F NR-RNs have been designed, where MIMO and out-band operation, i.e., the two links (backhaul and access links) have different work frequencies, are considered. Hence, the backhaul link works in the FR1 band. On the other hand, the access link operates in mmWave bands (FR2), which is achieved through the RF mmWave transmission chain and will be presented in the Section 3.2.

The 5G network scenario in Figure 4 is composed of one gNodeB, which will serve the one NR-UE through the NR-RN (A&F or D&F). We have assumed that the backhaul link is given by a Line-of-Sight (LOS) scenario, and the distance between gNodeB and NR-RN is approximately 67.0 m. In Reference [18], a large 5G NR number of propagation channel model that support frequency bands over the range 0.5–100 GHz, which can simulate environments as urban microcell street canyon, urban macrocell, indoor office, rural macrocell, and mobility, also including, a geometry-based stochastic channel model are presented. Specifically, for the simulations of the considered scenario, the Clustered Delay Line (CDL) model was implemented, as it is defined in Reference [18]. The CDL-D channel model is considered for the backhaul link, which is modeled according to the specification of the 3GPP. The 3GPP Urban Macrocell (Uma) pathloss model is used to represent the LOS backhaul link, whose expressions are given by

(10) $${\mathrm{PL}}_{\mathrm{BL}}\left(\mathrm{dB}\right)=\left\{\begin{array}{cc} {\mathrm{PL}}_{1}+\sigma_{\mathrm{BL}} & 10m\leq d_{\mathrm{BL}}^{1}\leq d_{\mathrm{BL}}^{†}\\ {\mathrm{PL}}_{2}+\sigma_{\mathrm{BL}} & d_{\mathrm{BL}}^{†}\leq d_{\mathrm{BL}}^{1}\leq 5\mathrm{km} \end{array}\right.,$$

where $d_{\mathrm{BL}}^{1}\left(m\right)$ is the distance from gNodeB to the NR-RN, as shown in Figure 4. Besides, $d_{\mathrm{BL}}^{†}$ (m) is the breakpoint distance, which can be calculated as Reference [18]; on the other hand, ${\mathrm{PL}}_{1}\left(\mathrm{dB}\right)$ and ${\mathrm{PL}}_{2}\left(\mathrm{dB}\right)$ are the path losses that can be given by

(11) $${\mathrm{PL}}_{1}\left(\mathrm{dB}\right)=28.0+22\log_{10}\left(d_{\mathrm{BL}}^{2}\right)+20\log_{10}\left(f_{c}\right),$$

and

(12) $${\mathrm{PL}}_{2}\left(\mathrm{dB}\right)=28.0+40\log_{10}\left(d_{\mathrm{BL}}^{2}\right)+20\log_{10}\left(f_{c}\right)-9\log_{10}\left({\left(d_{\mathrm{BL}}^{†}\right)}^{2}+{\left(h_{\mathrm{gN}}-h_{\mathrm{RN}}\right)}^{2}\right),$$

where $d_{\mathrm{BL}}^{2}\left(m\right)$ is the distance from the gNodeB antennas to the NR-RN Antennas, and $h_{\mathrm{gN}}$ and $h_{\mathrm{RN}}$ are the height of the gNodeB and NR-RN both in (m), respectively. $\sigma_{\mathrm{BL}}$ represents the shadowing term with zero mean and standard deviation of 6 dB, and fc is the carrier frequency in GHz.

The NR-RN process the received signals and they are then translated from FR1 bands to FR2 bands. The CDL-D channel model [18] and a LOS scenario indoor-to-indoor environment were considered in the Access Link. The 3GPP Indoor Office path loss model is employed to simulate the mmWave LOS Access Link, which is capable to capture various typical indoor deployment scenarios. The mmWave indoor path loss model can be expressed as

(13) $${\mathrm{PL}}_{\mathrm{AL}}=32.4+17.3\log_{10}\left(d_{\mathrm{AL}}^{1}\right)+20\log_{10}\left(f_{c}\right)+\sigma_{\mathrm{AL}}\;\;\;\;\;\;\left(1\;m\leq d_{\mathrm{AL}}^{1}\leq 100\;m\right),$$

where $d_{\mathrm{AL}}^{1}$ (m) represents the distance between NR-RN and NR-UE, and $\sigma_{\mathrm{AL}}$ denotes the shadowing term with zero mean and standard deviation of 3 dB. It should be noticed that both Backhaul Link and Access Link have used the same channel model, which is appropriated due to 3GPP recommendation [18], where, for the frequency range from 0.5 GHz to 100 GHz and LOS environments, the CDL-D can be employed. The difference is in the calculation of propagation losses. For the Backhaul Link and Access Link, expressions (10) and (13) should be applied, respectively.

### 3.1. D&F NR-RN Implementation

**Figure 5:**
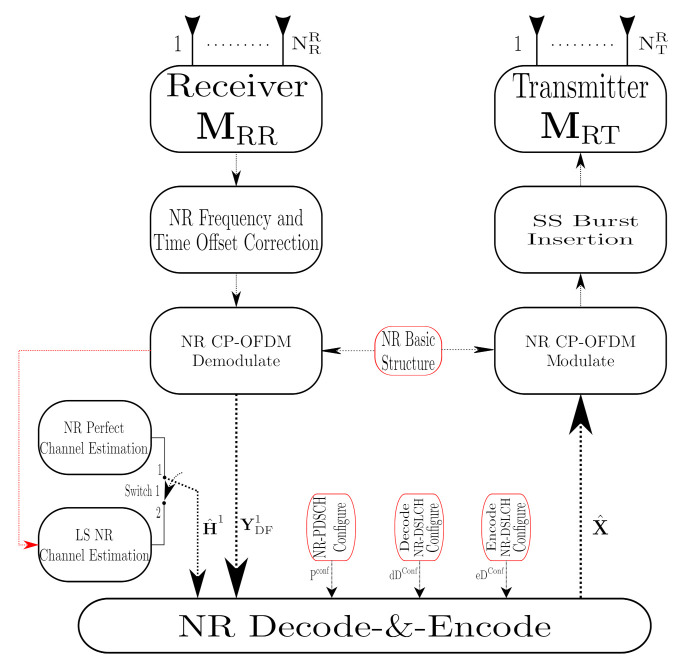
Decode-and-Forward (D&F) mmWave NR-RN processing blocks.

In this subsection, a NR-D&F protocol employing 5G communication ToolBox of MATLAB${}^{\mathrm{TM}}$ is presented. NR-D&F RN was implemented, where Frequency Division Duplexing (FDD-5G), multiple antennas, and out-band operation—that is, the two links (backhaul and access links) have different frequencies-are considered, which the Backhaul Link and Access Link work in FR1 and FR2, respectively. The implemented NR-D&F strategy estimates the channel or has the channel knowledge to perform received signal processing, depending on whether this functionality is activated, as can be seen in Figure 5.

From Figure 5, in the first-phase of the NR-D&F protocol, the IQ is captured with the Receiver block. After the acquisition of the data symbols, any significant frequency derivation must be estimated and eliminated, and the time offset must be performed, without which many errors would be propagated. After the steps described previously, the New Radio CP-OFDM is demodulated through the NR CP-OFDM Demodulate block, which is helped by a configuration structure, taking into account the sample rate of the received signal. In this regard, subcarrier spacing, cyclic prefix, the number of cell id, and the number of resource blocks are determined in the NR Basic Structure block. The first task after the received signal demodulation is to determine the channel, in which it should be noticed that the NR-D&F RN can be obtained two types of the channel by mean of two blocks (NR Perfect Channel Estimation and LS NR Channel Estimation blocks). In this context, the usage channel will depend on the selected point in switch 1. Thus, when point 1 is chosen the NR-D&F RN have knowledge of the channel, which in our case is the channel $H^{1}$ (CDL-D). However, when point 2 is selected the NR-D&F protocol performs a channel estimation through the LS NR Channel Estimation block, which is realized in two steps: first, the frequency responses for the subcarriers of the pilot symbols are determined, based on the Least Square (LS) estimator. On the other hand, from the first step, the frequency responses of the subcarriers of the data symbols can be derived by interpolation methods employing the adjacent pilot symbols.

Finally, the demodulated received signal ($Y_{\mathrm{DF}}^{1}$) and the estimated channel matrix (${\hat{H}}^{1}$) are processed through Algorithm 1 for the Decode-and-Encode of each subframe. Furthermore, for the proper functioning of Algorithm 1, the NR-PDSCH, Decode NR-DLSCH and encode NR-DLSCH configure blocks are token as input, which provide the parameter to decoding and encoding the PDSCH and DLSCH channels. This algorithm executes the decode and encode of the physical, control, and data channels, as well as determining other reference signals. It can be observed in Algorithm 1 that, in order to maximize the equalization Signal-to-Noise Ratio (SNR), the Mean Maximum Error square (MMSE) equalizer is employed. Besides, in the proposed algorithm, the sub-indices ${\left(\cdot \right)}^{d}$ and ${\left(\cdot \right)}^{c}$ in the functions describe the processes of decoding and encoding, as well as the sub-indices ${\left(\cdot \right)}^{b}$ and ${\left(\cdot \right)}^{s}$ in the outputs of the functions represent data bits and data symbols, respectively. The ${\mathrm{NR}-P}^{c}$ function, mapping each symbol, DMRS and PTRS signal in the resource grid, and the $\hat{x}$ subframe to the output of the function, is obtained. Finally, concatenating each subframe is performed by mean of the Cat(·) function, from which all frames to re-transmitting are saved. $\hat{X}$, the resultant signal to the output of Algorithm 1, is modulated through the NR CP-OFDM Modulate block. After that, an appropriated portion of SS Burst is added to the modulated signal. Then, the resultant waveform is passed by the mmWave transmitter $M_{\mathrm{RF}}$ block, which performs the translation in frequency (FR2) and transmits the signal to NR-UE, as will be presented in the following subsection.
**Algorithm 1:** Decoding-and-Encoding New Radio RN Algorithm.
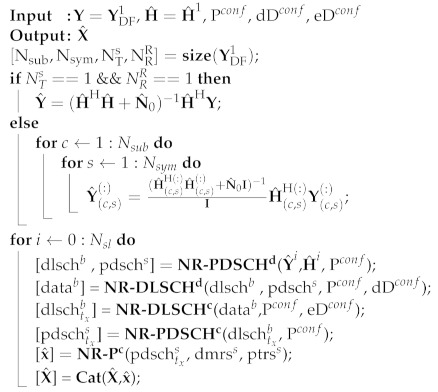


### 3.2. mmWave Transmitter and Receiver Chains Design

Today, most of the RF transceivers in wireless communications systems use the heterodyne architecture [19]. Therefore, in our design of the mmWave transmitter and receiver chains, we have opted for this type of architecture due to its excellent performance, as well as the fact that it is the only one that allows working with modulated signals in the mmWave frequencies and reduces the number of subsystems (mixers, amplifiers, filters, etc.) necessary to work in these frequency bands.

**Figure 6:**
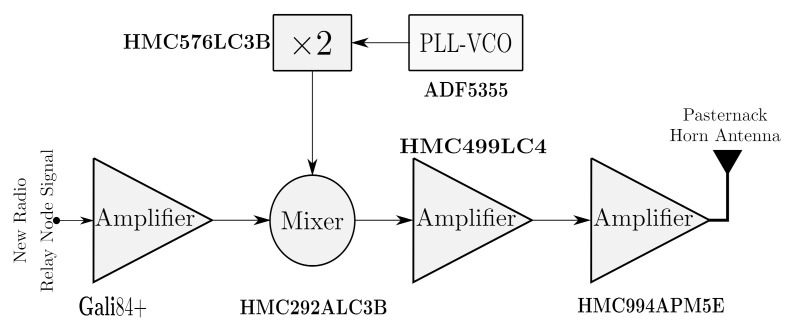
Subsystems of the mmWave transmitter chain (FR2−Tx).

A mmWave radio transmitter chain (FR2-Tx), which translates the signal from the FR1-Tx (<6 GHz) band to FR2 (mmW) band, after the application of the baseband algorithms, was designed. The signal in the mmWave frequency band will be transmitted through the radio channel and the received signal will be converted, through the mmWave radio receiver (FR2-Rx), into a baseband digital signal at the UE. The block diagram of mmWave transmitter chain is shown in Figure 6. For its implementation we have used off-the shelf subsystems. The last subsystem is Pasternack PE9851/2F WR-24 waveguide horn antenna with 20 dB gain [20], used at output of the mmWave transmitter chain. The horn antenna has a very directive radiation pattern which minimizes interference with other transmitters, allowing easy connection to the output power amplifier. For the mmWave medium power amplifier (MPA) and power amplifier (PA), Hittite HMC499LC4 [21] and Hittite HMC944APM5 [22] amplifiers were used. For the mmWave mixer, Hittite HMC292ALC3B [23] is employed. To generate local oscillator (LO) signals, we make the usage Analog Devices ADF5355 Phase-Locked Loop (PLL) [24] and Hittite HMC576LC3B frequency doubler [25]. The ADF5355 has integrated a wideband microwave VCO design permits frequency operation from 6.8 GHz to 13.6 GHz at one radio frequency (RF) output. This output frequency is multiplied by the frequency doubler to generate the LO signal. It is an optimal solution to reduce el number of mmWave components [26]. Once the modulation coding and baseband error correction algorithm were applied to FR1-Tx, the signal is applied to a Mini-Circuits Gali84 + amplifier [27] placed before mixer. The objective is to increase the output signal level and isolate it from the mixer load variations. The electrical specifications of the different RF subsystems are shown in Table 1.

**Figure 7:**
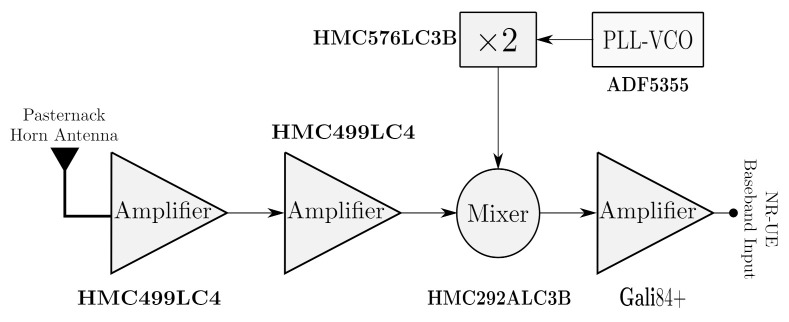
Subsystems of the mmWave receiver chain (FR2−Rx).

For the first stage of the NR-EU (FR2-Rx), a mmWave receiver chain was designed and implemented. Its block diagram, shown in Figure 7, has the same structure as transmitter chain and uses the same components, except that the power amplifier (PA) is replaced by a low noise amplifier (LNA). Specifically, the LNA from Hittite HMC519LC4 [28] was employed. The noise figure of this amplifier directly limits the sensitivity of the receiver. The electrical specifications of the low noise amplifier are shown in Table 1. As mentioned above, the platform was designed with connectorized off-the-shelf components, allowing for easy characterization and integration into the transceiver (Rx-Tx) mmWave radio platform. The cost of the mmWave transmitter and receiver chains is, approximately, 11,000$.

To support flexible development, we have designed our RF transmitter and receiver chains to allow easy integration with USRP radio software. This allows easy manipulation of the mmWs signals using our SDR platform developed for the implementation of Relay Nodos in the Research Group [29,30]. Simulink${}^{\mathrm{TM}}$ software of Mathworks was used to simulate the RF transmitter and receiver chains. This simulation software uses a power series model or polynomial model for nonlinear circuit modeling without memory. This model is widely used in the literature and is given by [31,32],

(14) $$V_{0}\left(t\right)=\sum_{k=0}^{K}C_{2k+1}{\left[V_{\mathrm{in}}\left(t\right)\right]}^{\left(2k+1\right)},$$

where K is nonlinear order, $V_{0}\left(t\right)$ is the output signal, $V_{\mathrm{in}}\left(t\right)$ is the input signal, and $C_{2k+1}$ is the (2k+1) the real polynomial coefficient.

According to the Simulink${}^{\mathrm{TM}}$ manual [33], the order of the nonlinearity will depend on the nonlinear parameters available for the different RF subsystems of the transmitting and receiving chain. In general, for all RF subsystems, we have: (a) the output third-order intercept point (OIP3), (b) the output power at 1 dB compression ($P_{1\mathrm{dB},\mathrm{out}}$), (c) the output power saturation ($P_{\mathrm{sat},\mathrm{out}}$), and (d) the gain compression at saturation (${\mathrm{GC}}_{\mathrm{sat}}$).

Under these conditions, we will have a nonlinearity of order K=7 and Equation (14) translates to:

(15) $$V_{0}\left(t\right)=C_{1}V_{\mathrm{in}}\left(t\right)+C_{3}V_{\mathrm{in}}^{3}\left(t\right)+C_{5}V_{\mathrm{in}}^{5}\left(t\right)+C_{7}V_{\mathrm{in}}^{7}\left(t\right),$$

and the coefficients $C_{1}$, $C_{3}$, $C_{5}$, and $C_{7}$ are calculated taking into account the following relationships:

(16) $$\begin{array}{cc} P_{\mathrm{sat},\mathrm{out}}+{\mathrm{GC}}_{\mathrm{sat}} & =P_{\mathrm{sat},\mathrm{in}}+G_{\mathrm{lin}}\\ P_{1\mathrm{dB},\mathrm{out}}+1 & =P_{1\mathrm{dB},\mathrm{in}}+G_{\mathrm{lin}}\\ \mathrm{OIP}3 & =\mathrm{IIP}3+G_{\mathrm{lin}}, \end{array}$$

where the units of the specified parameters are in dB or dBm, and $G_{\mathrm{lin}}$ is $C_{1}$ in units of dB, and solving the following system of equations:

(17) $$\begin{array}{cc} \sqrt{P_{\mathrm{sat},\mathrm{out}}} & =C_{1}\sqrt{P_{\mathrm{sat},\mathrm{in}}}+C_{3}{\left(\sqrt{P_{\mathrm{sat},\mathrm{in}}}\right)}^{3}+C_{5}{\left(\sqrt{P_{\mathrm{sat},\mathrm{in}}}\right)}^{5}+C_{7}{\left(\sqrt{P_{\mathrm{sat},\mathrm{in}}}\right)}^{7}\\ \sqrt{P_{1\mathrm{dB},\mathrm{out}}} & =C_{1}\sqrt{P_{1\mathrm{dB},\mathrm{in}}}+C_{3}{\left(\sqrt{P_{1\mathrm{dB},\mathrm{in}}}\right)}^{3}+C_{5}{\left(\sqrt{P_{1\mathrm{dB},\mathrm{in}}}\right)}^{5}+C_{7}{\left(\sqrt{P_{1\mathrm{dB},\mathrm{in}}}\right)}^{7}, \end{array}$$

where

(18) $$C_{1}=10^{\frac{G_{\mathrm{lin}}\left(dB\right)}{10}},$$

and

(19) $$|C_{3}|=\frac{C_{1}}{10^{\frac{\mathrm{IIP}3(\mathrm{dBm})}{10}}}$$

**Figure 8:**
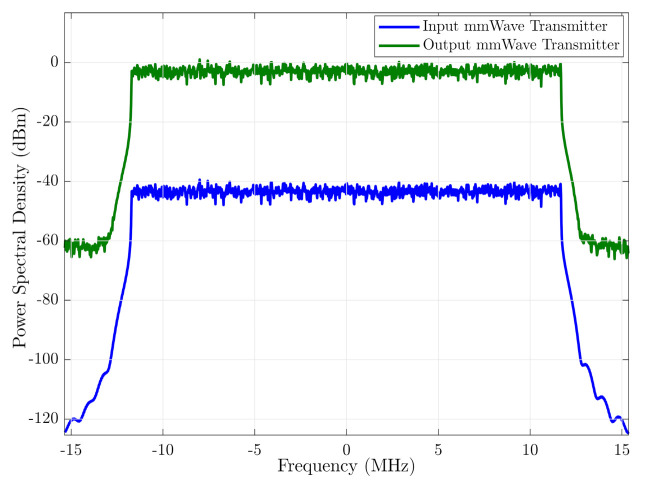
Spectrum of the input and output of the mmWave RF transmitter chain.

**Figure 9:**
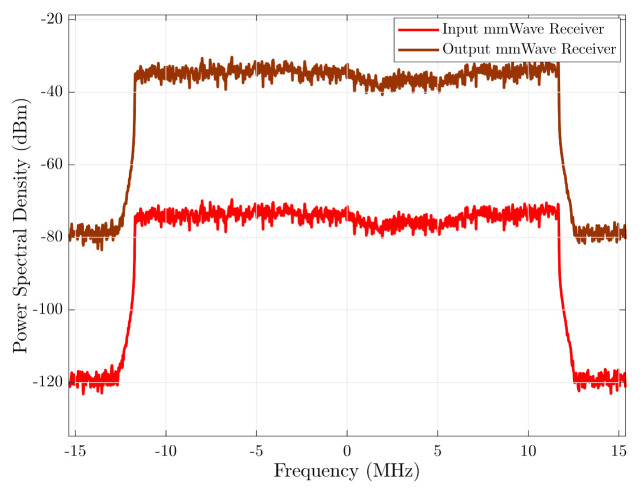
Spectrum of the input and output of the mmWave RF receiver chain.

The simulation of the input and output signal spectrum in the RF transmitter chain is shown in Figure 8. The input signal level was adjusted so that the amplifiers work in a linear zone and do not distort the output signal, preserving the shape of the input signal. As shown in the figure, the gain of the chain is approximately 40 dB. In the same way, Figure 9 shows simulation of the input and output signal spectrum in the RF receiver chain. The channel power gain variation of the simulated signals is due to the propagation model used in the simulation. The propagation model and the used parameters in the simulation were described in the corresponding sections.

## 4. Numerical Results and Discussion

In the section, the simulation results of the two-hop mmWave MIMO downlink RN architecture are presented. To assess the key performance indicators of the considered design, we present the results of Monte-Carlo computer simulations, aimed at BER and throughput versus Signal-to-Noise Ratio (SNR) of the proposed two-hop Relay cooperative system. We consider a network encompassing one gNodeB equipped with $N_{T}^{S}=\left\{1,2,4\right\}$ transmitter antennas, and transmitting 64-QAM and 256-QAM symbols. The RN (A&F or D&F) is equipped with $N_{R}^{R}=N_{T}^{R}=\left\{1,2,4\right\}$ receiver and transmitter antennas. Besides, the NR-UE is equipped of $N_{R}^{D}=\left\{1,2\right\}$ receiver antennas. Note that, when performing MIMO simulations, you will have as many transmitter and receiver chains as the number of antennas used in the simulation. The KPIs are evaluated by carrying out $10^{3}$ independent Monte-Carlo trials, which in each run is used independent sets of channel realizations and noise. For each SNR transmission, $10^{3}$ frames were sent, where the Relay processes the signal and forwards it. In Table 2, the simulation parameters are summarized, and the correspondent distances to calculate of the path loss in the scenario are shown in Table 3.

**Figure 10:**
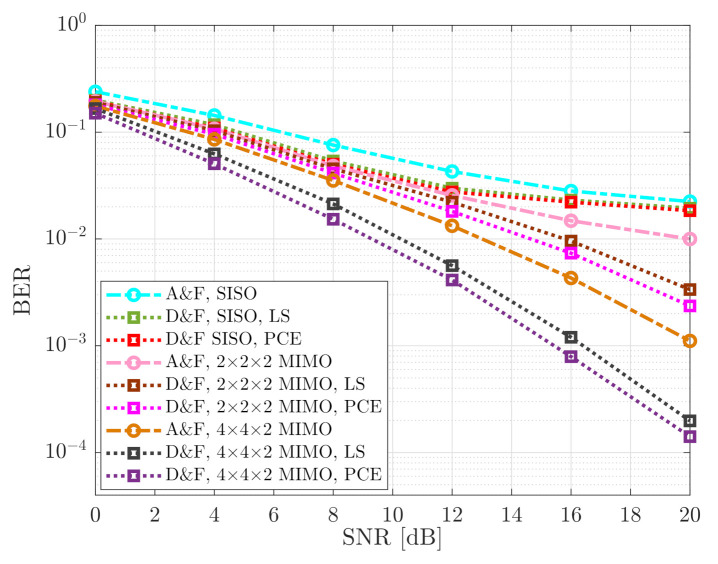
Bit Error Rate (BER) vs. Signal-to-Noise Ratio (SNR) of the cooperative system; 64-QAM signal.

Two types of NR-RNs are considered: A&F and D&F. In the case of the NR D&F RN two situations are developed: (a) an exact knowledge of the channel is available, and (b) an estimation of it is made. Furthermore, we have denoted $N_{T}^{S}\times N_{R}^{R}=N_{T}^{R}\times N_{R}^{D}$, like the considered MIMO scheme in our mmWave cooperative system. Figure 10 shows the corresponding BER performance comparison of the mmWave MIMO cooperative network of the implemented relaying strategies, considering the 64-QAM constellation. It may be seen that A&F protocol with SISO achieved a performance discrete. Besides, the D&F strategy with the knowledge of the channel (PCE) and SISO reached the best performance among the implemented NR-RN with SISO technology. However, the mmWave NR-D&F protocol with LS channel estimation presented a close BER in comparison when was considered the knowledge of the channel. From Figure 10, it can be verified that the D&F SISO with PCE reached the BER level of 1.8 × 10${}^{-2}$ at around SNR=20dB. In this context, the MIMO technique was introduced in the cooperative system, which specifically (2 × 2) or (4 × 4) MIMO and (2 × 2) or (4 × 2) MIMO in the Backhaul Link and Access Link, respectively, was considered. With the MIMO schemes, it can be seen that the cooperative system considerably increased the BER performance, in comparison with SISO technique. In this regard, we note that the (2 × 2 × 2) NR-A&F protocol achieves the same BER level of the D&F SISO with PCE at around SNR=14dB, so that a performance gain of 6 dB is obtained. Furthermore, when (4 × 4 × 2) NR-A&F strategy is taken into account, a BER level of 1.1 × 10${}^{-3}$ is reached, which supposes an enhancement of 7 dB in comparison with (2 × 2 × 2) NR-A&F.

**Figure 11:**
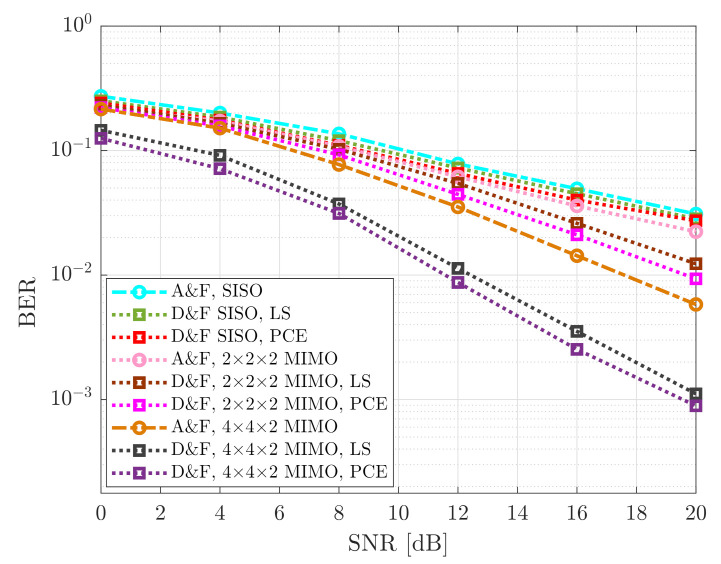
BER vs. SNR of the cooperative system; 256-QAM signal.

On the other hand, the NR-D&F strategy with MIMO, have generated an improved behavior. In this sense, it can be seen that the (2 × 2 × 2) NR-D&F strategy with LS channel estimation acquired a BER performance of 9.9×10${}^{-3}$ at around SNR=16dB, implying that 8 dB and 4 dB performance gain was successfully reached, in comparison with SISO NR-D&F and (2 × 2 × 2) NR-A&F strategies, respectively. Nevertheless, (4 × 4 × 2) NR-A&F strategy overcome the performance of the (2 × 2 × 2) NR-D&F protocol with PCE in approximately 2.5 dB. Besides, it can be noticed, from Figure 10, that the (4 × 4 × 2) NR-D&F protocol with knowledge of the channel generally achieved a better BER level of the proposed algorithms, more specifically, obtained the same performance as the (4 × 4 × 2) NR-D&F with LS channel estimation at SNR=19.2dB, such that an 0.8 dB performance gain was attained and overcoming the (4 × 4 × 2) NR-A&F strategy from SNR=15.5dB. We also have performed other curves for comparison of the mmWave MIMO cooperative system with the proposed schemes, which were presented from Figure 11; in this case, the 256-QAM constellation was considered. From reported BER in Figure 11, we can be observed that the developed relaying strategies present the same behavior. Nevertheless, it should be noticed that BER performance of the 64-QAM is better than 256-QAM, which was achieved due to the robustness that presents the 64-QAM front to 256-QAM. For example, it can be verified that the (4 × 4 × 2) NR-D&F strategy with 64-QAM scheme achieved reaching the same BER performance of the (4 × 4 × 2) NR-D&F protocol with 256-QAM constellation at around SNR=15.8dB, which supposes a performance gain of 4.2dB.

**Figure 12:**
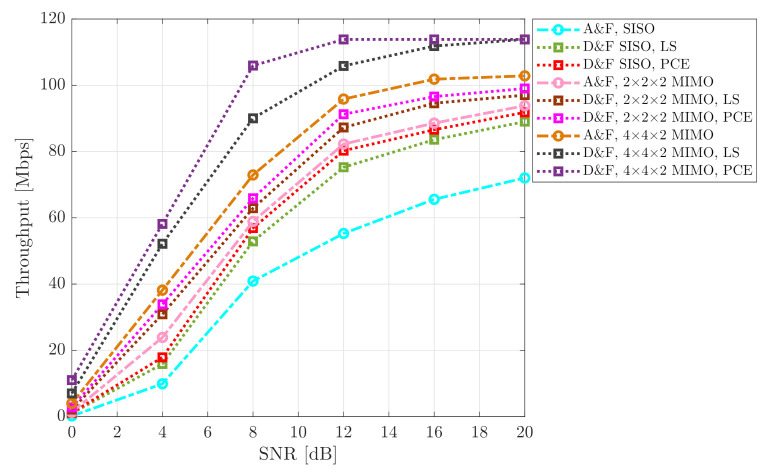
Throughput vs. SNR of the cooperative system; 64-QAM signal.

**Figure 13:**
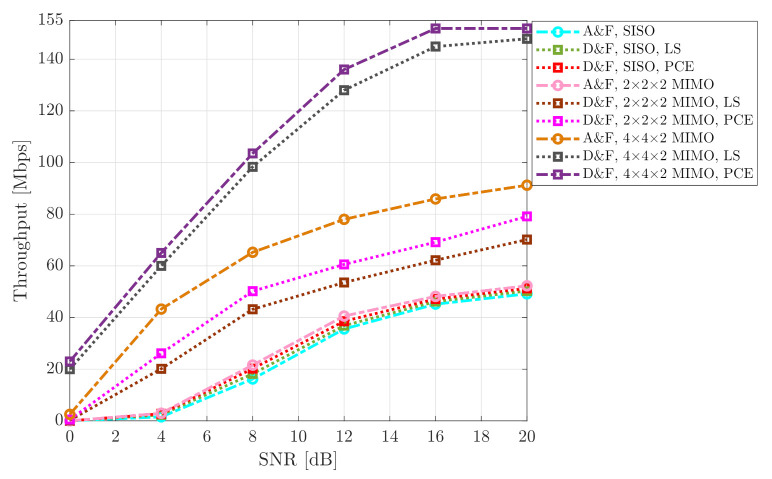
Throughput vs. SNR of the cooperative system; 256-QAM signal.

To evaluate the behavior of the implemented architectures, another KPI was considered. The output throughput of the processing of the New Radio DL-SCH transport channel was measured. The achievable throughput recorded for SISO and MIMO cooperative system for various mmWave NR-RN protocols are shown in Figure 12 and Figure 13, for the 64-QAM and 256-QAM constellations, respectively. It can be observed, from the figures that the SISO D&F with LS technique achieved higher average throughput than the SISO A&F strategy. However, it may also be seen that, when the SISO NR-D&F with PCE scheme is introduced, the achievable capacity of the system is improved in comparison with the architectures mentioned above. More particularly, in the 64-QAM case for mmWave NR-D&F without channel knowledge, from Figure 12, indicates that the throughput at SNR=20dB was approximately 89.03Mbps; nevertheless, with channel knowledge, a throughput of 91.83Mbps could be reached with the same SNR value, such that a performance gain of about 17.00Mbps and 19.80Mbps, respectively, in comparison with 72.03Mbps achieved by SISO A&F architecture. On the other hand, when compared with mmWave (2 × 2 × 2) NR-RN strategies, the achieved capacity is much higher than the SISO NR-RN protocols, as can be seen in Figure 12 and Figure 13. In this context, the maximum performance of the SISO mmWave cooperative system was reached by the mmWave (2 × 2 × 2) MIMO NR-A&F at SNR=19dB, which has introduced an improved the system performance of around 2.1Mbps, for 64-QAM constellation. Besides, it should be noted that (2 × 2 × 2) NR-D&F protocol with LS and PCE have obtained 97.00Mbps and 99.13Mbps, respectively. From Figure 13, it can be noted that the maximum throughput of the mmWave SISO cooperative network was achieved from SNR=6.5dB, such that a capacity gain of about 28.16Mbps was obtained when the (2 × 2 × 2) MIMO NR-D&F protocol with PCE was considered.

It worth noting that the impact of decoding and encoding steps that the received signal is subjected to before being re-transmitted to the NR-UE by the NR-D&F protocols are noticed when the MIMO technique is considering. Therefore, we also have presented the throughput of the mmWave (4 × 4 × 2) MIMO NR-D&F strategies, as are shown in Figure 12 and Figure 13. Furthermore, it can be seen that, when NR-D&F with channel knowledge is employed, the better capacity between the considered cooperative systems is achieved, for both 64-QAM and 256-QAM constellations. However, when mmWave (4 × 4 × 2) NR-D&F with LS channel estimation is considered, the achievable throughput is nearest to the reached with channel knowledge. Furthermore, it should be noticed that the maximum performances of our system are 113.85Mbps and 151.90Mbps, which were reached by mmWave (4 × 4 × 2) NR-D&F from SNR=12dB and SNR=16dB for 64-QAM and 256-QAM cases, respectively. Finally, the achieved maximum performance by mmWave (4 × 4 × 2) NR-D&F protocol with the 256-QAM constellation was 38.05Mbps higher than the 64-QAM scheme.

### Comparative Discussion

In this subsection, the presented architecture is compared with previous works evaluating similar architectures [34,35,36,37], in terms of the type of implemented RNs in the architecture, the working frequency of the backhaul and access links, and if the architecture under analysis was implemented by hardware, as shown in Table 4. In general, in these works, the operation of the A&F and D&F protocols were analyzed and compared, showing that the use of D&F RNs presents an improvement in the performance of the architecture compared to A&F RNs. A limitation found in the analyzed studies is the considered signal model. In general, in the analyzed studies, the considered signal model is not a signal model standardized by the 3GPP [16], as proposed in this work. This paper also proposes a hardware implementation of the relay nodes through the design of mmWave transmitter and receiver chains with off-the shelf, having considered in the simulations the non-linearities of the used subsystems. On the other hand, our proposal has the disadvantage of working in Half Duplex mode, compared to the Full Duplex operation of the other architectures considered [34,35,36,37], which will be addressed in further research.

## 5. Conclusions

In this paper, a two-hop 5G New Radio architecture was studied and analyzed in order to enhance the average system throughput and BER in outdoor-to-indoor environments. The system implements an NR-RN cooperative network, which uses outband NR-RNs. These NR-RNs translate the incoming signal into mmWave band of the 26 GHz for the access link inside an office environment. Two protocols of RNs were analyzed: Amplify-and-Forward (A&F) and Decode-and-Forward (D&F). Moreover, the channel estimation stage for the D&F relay was considered, taking into account the knowledge of the channel and applying the channel estimation through Least Square (LS) estimator. The cooperative system deploys NR-RN with one antenna at transmission and one antenna at reception, as well as multiple antennas. Furthermore, the 5G NR signal employs features that are standardized by the 3GPP.

In the simulations, the results demonstrated that the D&F NR-RN scheme can greatly enhance the KPIs of the system in comparison with A&F protocol. From the results, it follows that the implemented D&F NR-RN protocol with knowledge of channel has the best performance. For example, the maximum throughput of the proposed architecture (113.85 Mbps for 64-QAM signal) is reached from SNR=12dB. Nevertheless, with channel estimation, the obtained results are very near to the best performance, which has achieved approximately 93% of the maximum throughput at the SNR=12dB for the 64-QAM scheme. It can be noticed that is excellent results due to that, in a real environment, the channel needs to be estimated. On the other hand, the results showed that the 64-QAM modulation leads to better robustness in the evaluated scenario. Furthermore, it can be concluded from the results that employing mmWave NR-RNs together MIMO technique led to a substantial benefit, in terms of the KPIs of the network in the outdoor-to-indoor environment. The MIMO scheme achieved a reduction of the BER and increasing the throughput in the 256-QAM modulation scheme, which can be concluded increasing the number of antennas can lead to higher performance and spectral efficiency of the system.

## Figures and Tables

**Table 1:** Parameters of the elements of mmWave transmitter and receiver chains.

Parameters	Gali${}_{84}$	HMC292ALC3B	HMC499LC4	HMC944APM5E	HMC519LC4
Frequency Range (GHz)	0–6	14–30	24–28	20–28	18–28
$G_{\mathrm{lin}}$ (dB)	19.2	–9	16	16	14.4
Local Oscillator Frequency (GHz)	-	25	-	-	-
Input Return Loss (dB)	18	13.2	8	12	15
Output Return Loss (dB)	8.9	12.5	12	22	20
OIP3 (dBm)	38	20	34	36	23
$P_{1\mathrm{dB},\mathrm{out}}$ (dBm)	21.2	9	23	27	11
$P_{\mathrm{sat},\mathrm{out}}$ (dBm)	22.1	13	23.5	28	14
Noise Figure (dB)	4.4	10	5	4	3.5
${\mathrm{GS}}_{\mathrm{sat}}$ (dB)	3.5	2.2	4	5	6

**Table 2:** Main parameters of simulation.

Parameters	Backhaul Link	Access Link
Signal Bandwidth	25 MHz	25 MHz
Carrier Frequency	3.5 GHz	26.1 GHz
Tx/Rx schemes	SISO, 2 × 2 and 4 × 4	SISO, 2 × 2 and 4 × 2
SSB Configuration	Case B	Case B
transmitted SSB	4 blocks	4 blocks
Subcarrier Spacing	30 kHz	30 kHz
SSB periodicity	20 ms	20 ms
Modulations	64-QAM and 256-QAM	64-QAM and 256-QAM
Channel Models	CDL-D	CDL-D
Light-Vision	LOS	LOS

**Table 3:** Distance of the simulation scenario.

Parameters	Values
$h_{\mathrm{gN}}$	25 m
$h_{\mathrm{RN}}$	19.5 m
$d_{\mathrm{BL}}^{1}$	67 m
$d_{\mathrm{BL}}^{2}$	67.3 m
$d_{\mathrm{AL}}^{1}$	6.88 m

**Table 4:** A comparison survey with previous works.

Parameters	[34,35]	[36]	[37]	Proposed
Signal Model	Conventional	Conventional	Conventional	NR standardization
A&F RN	No	Yes	Yes	Yes
D&F RN	Yes	No	No	Yes
Number of RN	1	4	1	1
Duplex Mode	Full	Full	Half	Half
BL Frequency	60 GHz, 28 GHz	<6 GHz	<6 GHz	3.5 GHz
AL Frequency	60 GHz, 28 GHz	<6 GHz	<6 GHz	26 GHz
Channel Estimation Algorithm	No	No	Yes	Yes
Knowledge Channel	Yes	Yes	Yes	Yes
Proposal of Hardware Implementation	No	No	No	Yes

## Data Availability

Data sharing not applicable.
